# Supplementary material for: PLEKHM1 Overexpression Impairs Autophagy and Exacerbates Neurodegeneration in rAAV-α-Synuclein Mice
Source: Cells. 2025 Aug 29;14(17):1340. doi: 10.3390/cells14171340 (PMC12427628; doi:10.3390/cells14171340)
Supplement: Supplementary file 1 [file cells-14-01340-s001.zip › cells-3718532-supplementary.pdf]

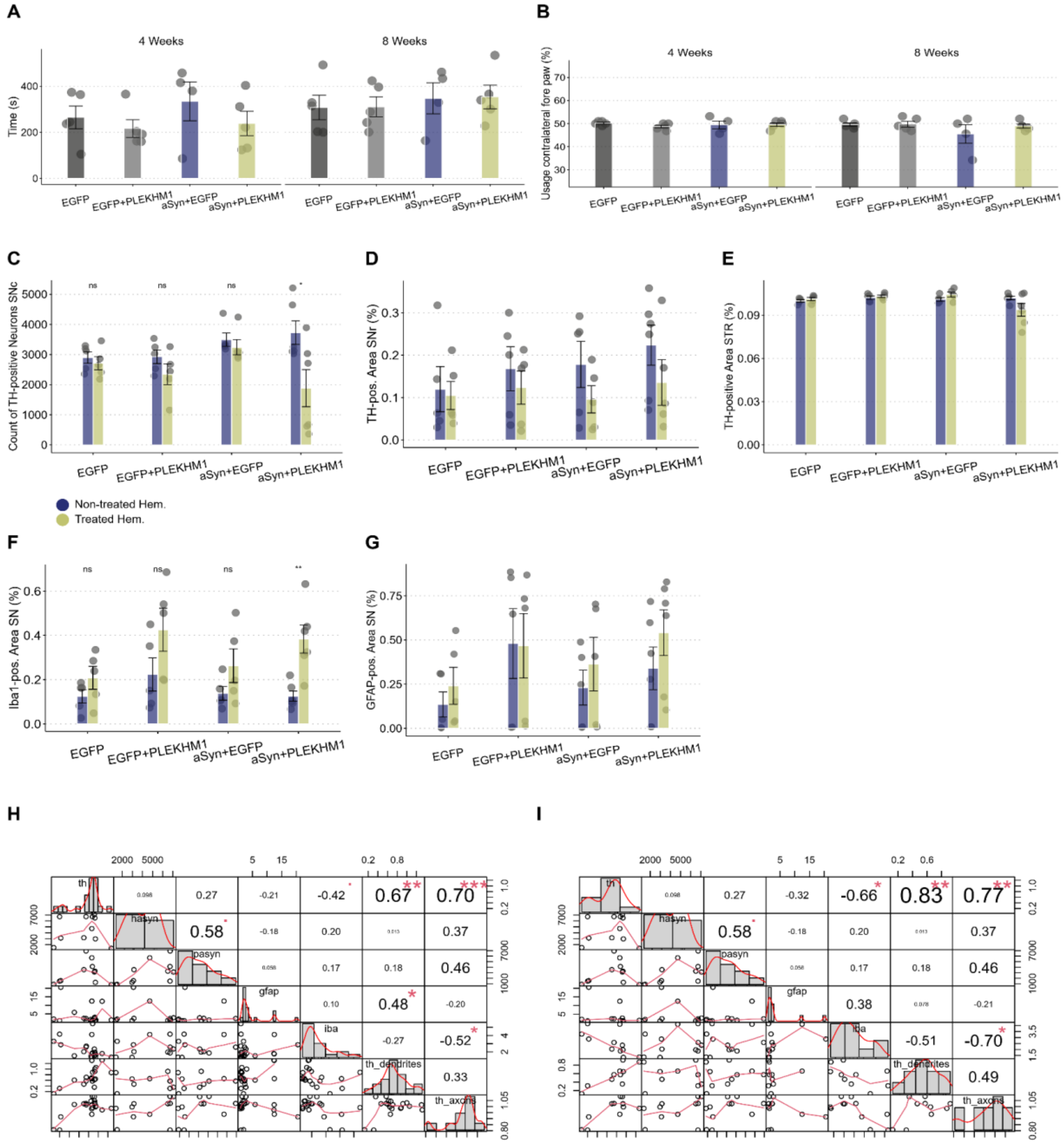

Supplementary Figure S1. A) The cylinder test was performed at two time points: four weeks and eight weeks after surgery. One animal at a time was transferred into a 15-cm-wide glass cylinder and up to 25 contacts with the cylinder wall were videotaped. The time until the dominant forepaw performed 25 contacts was scored based on the videos. B) Forepaw use was assessed by calculating the ratio of left (potentially impaired) to right forepaw contacts during the cylinder test. This provided a measure of asymmetry in spontaneous limb use based on the number of wall contacts made by each forepaw. C) Absolute count of dopaminergic neurons in the SNc of both hemispheres, providing the original, non-normalized values underlying the normalized data presented in Figure 2B. D) TH immunoreactivity in the SNr, quantified as the TH-positive area relative to the total SNr area in both hemispheres. These raw values correspond to the normalized expression shown in Figure 2C. E) TH immunoreactivity in the STR of both hemispheres, representing the original measurements that support the normalized data illustrated in Figure 2E. F) Iba1-positive area in the

SNc, quantified as the Iba1-positive area relative to the total SNc area in both hemispheres, reflecting microglial activation prior to normalization, as presented in Figure 3B. G) GFAP-positive area in the SNc of both hemispheres, representing astrocytic activation prior to normalization, as shown in Figure 3E. H) Correlation matrix including all animals across experimental groups, based on normalized values of histological measures. The matrix displays Pearson correlation coefficients and with statistical significance indicated as follows: $p < 0.05$ (*), $p < 0.01$ (**), and $p < 0.001$ (***). I) Correlation matrix restricted to αSyn-expressing animals, using the same data. This subgroup analysis highlights correlations specific to the aSyn condition, with R-values and statistical significance as indicated above.
